# Variations in the Position and Length of the Vermiform Appendix in a Black Kenyan Population

**DOI:** 10.1155/2014/871048

**Published:** 2014-04-30

**Authors:** Philip Mwachaka, Hemed El-busaidy, Simeon Sinkeet, Julius Ogeng'o

**Affiliations:** Department of Human Anatomy, University of Nairobi, P.O. Box 30197, Nairobi 00100, Kenya

## Abstract

*Background.* Topography of the appendix influences its mobility, degree of mobilization of the cecum, and need for additional muscle splitting during appendectomy. Although appendectomy is a common surgical procedure, there is a paucity of data on its topography in black Africans. * Methods.* The position and length of the appendix and relation of the appendicular base with spinoumbilical line were determined in 48 cadavers obtained from the Department of Human Anatomy, University of Nairobi, Kenya. *Results.* The commonest appendicular types in males were retrocecal 10 (27%) while in females was subileal 4 (36.4%). The average length of the appendix was 76.5 ± 23.6 mm. The base of the appendix was located along, below, and above the spinoumbilical line in 25 (52.1%), 9 (18.8%), and 14 (29.2%) cases, respectively. *Conclusion.* The topography of appendix in Kenyans shows variations from other populations. Knowledge of these variations is important during appendicectomy.

## 1. Introduction

The vermiform appendix is the most variable abdominal organ in terms of position, extent, peritoneal, and organ relations [1–4]. Knowledge of the variations in the position of the vermiform appendix is important because, in appendicitis, its variable positions may produce variable symptoms and signs which mimic other diseases [5–7]. Further, understanding of these variations is important during other intra-abdominal procedures [4, 8]. The length of the vermiform appendix is important in influencing the differential diagnosis of acute abdomen [2].

Ethnic and geographical variations have been reported regarding the position of the appendix (Table 1). This variable anatomy may pose a challenge during appendectomy because it may necessitate extension of a transverse incision or additional muscle splitting. Both these may complicate the surgery, prolong the operating time, and can affect the cosmetic outcome [9]. Awareness of these variations is therefore important for preoperative planning. Although appendectomy still remains one of the most commonly performed surgical procedures in Kenya [10–12], there is still scarcity of data on variant anatomy of the vermiform appendix in Kenyans. This study therefore aimed to investigate the topography of the appendix in a black Kenyan population.

## 2. Materials and Methods

**Figure 1:**
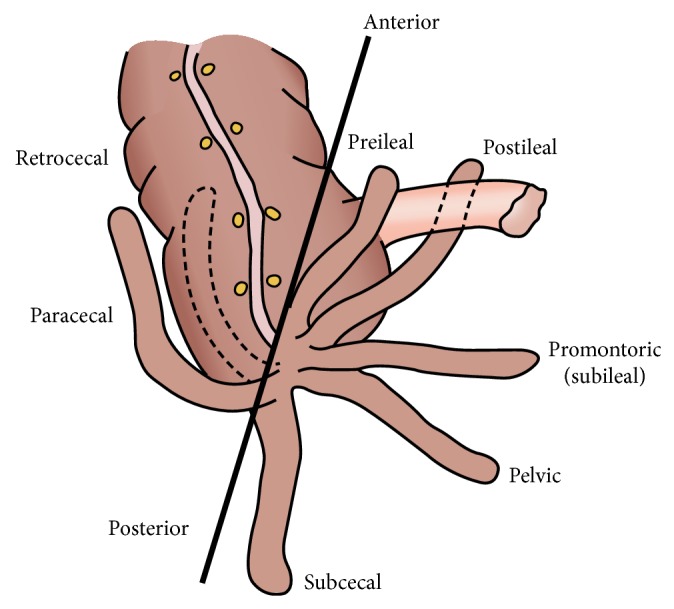
Scheme showing various vermiform appendix positions (Adapted from O'Connor and Reed [13]).

Forty-eight human cadavers (37 males) obtained during routine dissection in the Department of Human Anatomy, University of Nairobi, were studied. Ethical approval was obtained from the Kenyatta National Hospital/University of Nairobi Ethics and Review Committee before commencement of the study. Subjects with any gross abnormalities of abdominal organs, fibrosis, kinking or adhesions, and history of abdominal surgery were excluded. Following resection of the anterior abdominal wall, the position of the base of vermiform appendix was determined using the schema derived by [13] (Figure 1). Representative photographs were taken using a Fujifilm A235 digital camera.

Measurement of the length of appendix from its base was taken using a string and a ruler. The distance from anterior superior iliac spine (ASIS) and umbilicus (the spinoumbilical line) was measured. McBurney's point was taken to be the proximal two-thirds of the spinoumbilical line. The relation of the base of the appendix with McBurney's point was determined and classified as cephalad, caudad, or along the spinoumbilical line. Data collected was coded and entered in Microsoft Office Access and analyzed using SPSS for Windows version 18.0 (Chicago, Illinois).

## 3. Results

### 3.1. Position of the Base of the Appendix

**Figure 2:**
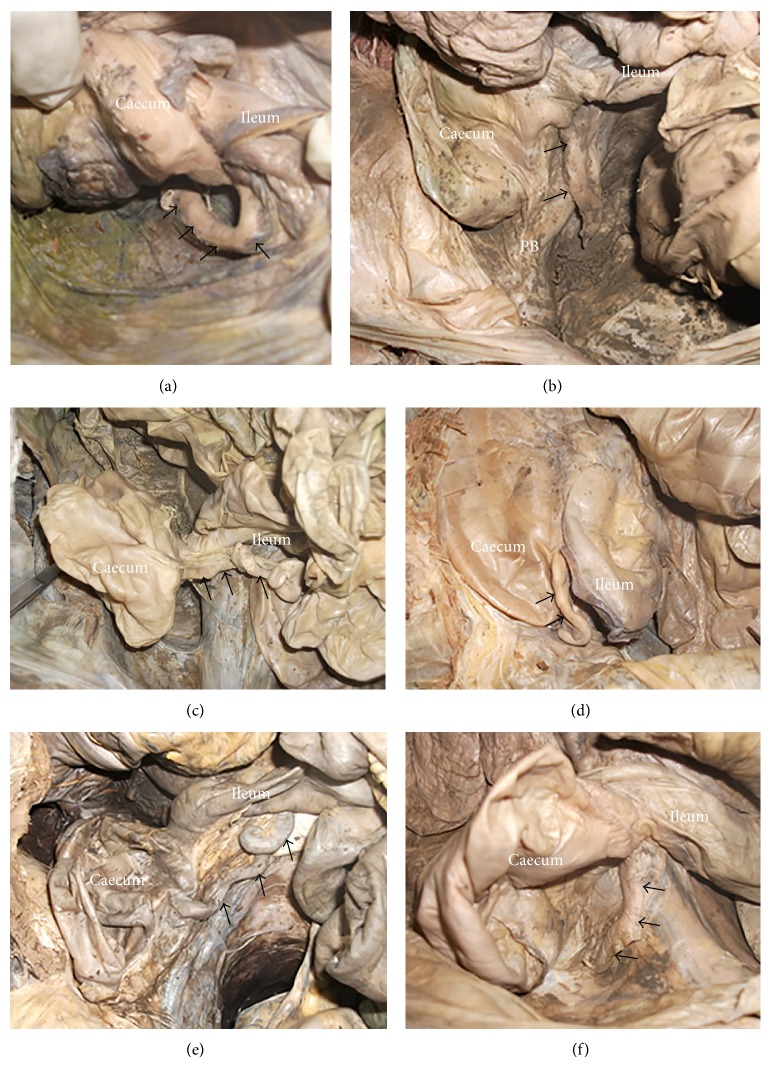
Position of the appendix (arrow). (a) Retrocecal appendix (note the appendix curving behind the cecum). (b) Pelvic appendix (note the appendix crossing the pelvic brim (PB)). (c) Preileal appendix. (d) Subileal appendix. (e) Postileal appendix. (f) Subcecal appendix.

The most common position of the appendix overall was retrocecal (Figure 2(a)), followed by the pelvic type (Figure 2(b)). Other variations seen include preileal (Figure 2(c)), subileal (Figure 2(d)), postileal (Figure 2(e)), and subcecal type (Figure 2(f)).  Table 2 summarizes these results. The commonest types in males were retrocecal 10 (27%) and pelvic 10 (27%), while in females it was subileal 4 (36.4%).

### 3.2. Length of the Appendix

The average length of the appendix was 76.5 ± 23.6 mm, with a minimum of 35 mm and maximum of 145 mm.  Table 2 summarizes the lengths of the appendices based on their anatomical position. The longest appendix was paracecal type (110.0 mm), while the shortest was subhepatic (63.0 ± 32.5 mm).

### 3.3. Relation to Spinoumbilical Line

The average distance between the anterior superior iliac spine and umbilicus (spinoumbilical line) was 158.3 ± 17.9 mm, with a minimum of 130 mm and a maximum of 200 mm. The base of the appendix was located along the spinoumbilical line in 25 (52.1%) cases. In the remaining half it was not located along the spinoumbilical line. In 9 (18.8%) cases it was below and medial to the line, and in 14 (29.2%) cases it was above and lateral to this line.

For appendices that were located along the spinoumbilical line, the average distance from the anterior superior iliac spine to the base of the appendix was 83.9 ± 11.5 mm, and thus most appendices were on average located approximately at the midpoint of spinoumbilical line and not at the popular Mc Burney's point. For appendices located below and above the spinoumbilical line, they were 88.0 ± 13.0 mm and 100 ± 17.3 mm far from the anterior superior iliac spine, respectively (Table 3).

## 4. Discussion

The classic teaching in many surgical training centers is that the appendix lies deep at the junction between the lateral and middle thirds of the right spinoumbilical line, so-called Mc. Burney's point [17]. However, in the current study, 48% of appendicular bases were not along the spinoumbilical line. This finding is clinically significant. In Africa where open appendectomies form the significant majority [11, 18, 19], surgeons need to be aware of this variation for preoperative planning and better surgical outcomes. Current results postulate that trainee surgeons should not be surprised if the appendix is not easily visualized when a transverse incision is made at the McBurney's point.

A remarkable finding of the present study was that, of the 48% appendices that were not along the spinoumbilical line, approximately 30% were cephalic to this line and furthest from anterior superior iliac spine (ASIS). Naraynsingh et al. [9], using a double contrast postevacuation barium enemas for evaluating the Mc. Burney's point, found that, for appendices that were cephalic to Mc. Burney's point, their average distance from ASIS was 42 mm [9]. Our study found an average distance of 100 mm, which is twice the previous study. This finding is clinically important because if the appendix is cephalic, access to the cecum becomes considerably more difficult when a transverse incision is made at the Mc. Burney's point [9]. It means surgeons in the study population may not find it uncommon to extend their incisions cephalad and do additional muscle splitting to locate the appendicular base. Our findings also concur with Ramsden et al. [20] from UK who found 15% of appendices were more than 10 cm from ASIS [20].

A study by D. Hegde and S. D. Hegde (2008), using 100 patients in whom a radio-opaque marker was placed during appendectomy, found a more superomedial location of the appendix in 75% of cases [21]. The study by Naraynsingh et al. also found 67% prevalence of appendices that were cephalic to spinoumbilical line [9]. However, other studies found a more caudal location of the appendix in their populations. Ramsden et al. from UK, for instance, found a more caudal position of the appendix in 75% of cases. Our study found prevalence of only 19% [20]. This difference may be due to ethnic variations in the position of the appendix.

The location of the appendix is important when it comes to clinical presentation of a patient with appendicitis. The area of tenderness in appendicitis will depend upon the length, position of the appendix, part of the appendix with inflammation, direction of the appendix, presence of fibrosis, and kinking or adhesions [1, 22]. In the current study, most appendices were retrocecal (27%) followed by pelvic (25%). Our results are concordant with a similar African study from Ghana, which found retrocecal prevalence of 67% [16]. A study among Indians also found a predominant retrocecal position in 68% of cases [23]. However, another African study from Zambia [24] found a predominant pelvic position (43.6%). These differences may be due to genetic and lifestyle factors like nutritional regimens [14].

The retrocecal position of the appendix is worth appraising. Retrocaecal appendicitis lacks distinctive clinical pattern and has been theorized to follow a more insidious course than other anatomic variants [17, 25]. There is often limited systemic upset and no progression to affect the general peritoneal cavity. In retrocaecal appendicitis it is difficult to elicit tenderness on palpation in the right iliac region and even deep pressure may fail to elicit tenderness because the caecum, distended with gas, prevents the pressure exerted by the palpating hand from reaching the inflamed appendix, so it has been termed “silent appendicitis” [22]. Retrocecal appendix has also been postulated to have high chances of gangrenous complication because their blood supply is more prone to kinking and more liable to inflammation when fixed retrocecally [26].

Two studies looked at the retrocecal position of the appendix and its influence on clinical presentation. Stranding, found no distinctive clinical pattern in a series of 105 cases [27]. The study by Herscu et al., which looked at retrocecal anatomy and perforation rates at presentation, also found no significant association between retrocecal position and perforation rates [25]. However, the risk of perforation was 60% higher in the retrocecal group. Comparing these two studies with previous ones, further research is needed to definitively quantify the clinical relevance of retrocecal appendix.

Subhepatic location of the appendix is generally rare [28], with most cases being documented in case reports. A notable observation in the present study was the relatively high frequency of subhepatic appendix (4.2%), only comparable to 4% reported among Pakistani's [15]. This position is thought to be caused by defective migration of the caecum during development or due to adhesions [7, 29, 30]. Knowledge of this position is important because subhepatic appendicitis can cause a diagnostic dilemma as it may mimic hepatobiliary or renal disease [7, 29, 30].

Mean length of the appendix in our study was 7.65 cm, within the range reported in the literature [4, 23, 24]. When inflamed, abnormally longer appendices may simulate inflammation of other structures such as enteritis, salpingitis, scrotal pains, and endometriosis [31–33]. Accordingly, appendicitis should always be considered as a differential diagnosis in acute abdomen even when the pattern of pain or tenderness is not at the right iliac fossa.

## 5. Conclusion

The topography of vermiform appendix in Kenyans shows variation from other populations. In a striking 30% of cases, the base of the appendix was cephalic to the McBurney's point and furthest from anterior superior iliac spine. This means surgeons employing transverse incisions may need to do additional muscle splitting to locate the appendicular base. In this part of the world where open appendectomies are common, surgeons need to be aware of this variation for better operative outcomes.

## Figures and Tables

**Table 1:** Positions of appendix in various populations.

Population	*N*	Position of the appendix (%)				
		Retrocecal	Pelvic	Postileal	Subcecal	Preileal
Croatian [1]	50	38	26	—	8	—
Bangladesh [3]	60	65	31.7	3.3	—	—
Iranian [14]	400	—	55.8	12.5	19	4.2
Pakistani [15]	500	57	28.6	9.4	—	4.0
Ghanaian [16]	1358	67.3	21.6	3.8	—	4.9

**Table 2:** Position and length of the vermiform appendix among males and females.

Position	Males (*n*)	Females (*n*)	Total *n* (%)	Mean length, mm	Std. deviation
Retrocecal	10	3	13 (27.1)	70.2	22.6
Pelvic	10	2	12 (25.0)	78.3	21.9
Postileal	8	1	9 (18.8)	87.1	29.1
Subileal	5	4	9 (18.8)	76.1	18.1
Subhepatic	2	0	2 (4.2)	63.0	32.5
Subcecal	1	1	2 (4.2)	70.0	42.4
Paracecal	1	0	1 (2.1)	110.0	

Total	37	11	48 (100)		

**Table 3:** Distance from the anterior superior iliac spine to the base of the appendix.

Appendix in relation SUL	*N*	Mean (mm)	Std. deviation
Above SUL	7	100.0	17.3
Along SUL	14	83.9	11.5
Below SUL	5	88.0	13.0

Total	26	89.0	14.7

SUL: spinoumbilical line.
